# Influence of biofilm growth age, media, antibiotic concentration and exposure time on *Staphylococcus aureus* and *Pseudomonas aeruginosa* biofilm removal in vitro

**DOI:** 10.1186/s12866-020-01947-9

**Published:** 2020-08-24

**Authors:** Xiaofeng Chen, Trine Rolighed Thomsen, Heinz Winkler, Yijuan Xu

**Affiliations:** 1 Center for Microbial Communities, Aalborg University, Aalborg East, Denmark; 2 Life Science Division, Danish Technological Institute, Aarhus, Denmark; 3 Osteitis Centre, Privatklinik Döbling, Vienna, Austria

## Abstract

**Background:**

Biofilm is known to be tolerant towards antibiotics and difficult to eradicate. Numerous studies have reported minimum biofilm eradication concentration (MBEC) values of antibiotics for many known biofilm pathogens. However, the experimental parameters applied in these studies differ considerably, and often the rationale behind the experimental design are not well described. This makes it difficult to compare the findings. To demonstrate the importance of experimental parameters, we investigated the influence of biofilm growth age, antibiotic concentration and treatment duration, and growth media on biofilm eradication. Additionally, OSTEOmycin™, a clinically used antibiotic containing allograft bone product, was tested for antibiofilm efficacy.

**Results:**

The commonly used Calgary biofilm device was used to grow 24 h and 72 h biofilms of *Staphylococcus aureus* and *Pseudomonas aeruginosa*, which were treated with time-dependent vancomycin (up to 3000 mg L^− 1^) and concentration-dependent tobramycin (up to 80 mg L^− 1^), respectively. Two common bacteriological growth media, tryptic soy broth (TSB) and cation-adjusted Mueller Hinton broth (CaMHB), were tested. We found for both species that biofilms were more difficult to kill in TSB than in CaMHB. Furthermore, young biofilms (24 h) were easier to eradicate than old biofilms (72 h). In agreement with vancomycin being time-dependent, extension of the vancomycin exposure increased killing of *S. aureus* biofilms. Tobramycin treatment of 24 h *P. aeruginosa* biofilms was found concentration-dependent and time-independent, however, increasing killing was indicated for 72 h *P. aeruginosa* biofilms. Treatment with tobramycin containing OSTEOmycin T™ removed 72 h and 168 h *P. aeruginosa* biofilms after 1 day treatment, while few 72 h *S. aureus* biofilms survived after 2 days treatment with vancomycin containing OSTEOmycin V™.

**Conclusions:**

This study demonstrated biofilm removal efficacy was influenced by media, biofilm age and antibiotic concentration and treatment duration. It is therefore necessary to taking these parameters into consideration when designing experiments. The results of OSTEOmycin™ products indicated that simple in vitro biofilm test could be used for initial screening of antibiofilm products. For clinical application, a more clinically relevant biofilm model for the specific biofilm infection in question should be developed to guide the amount of antibiotics used for local antibiofilm treatment.

## Background

To improve diagnosis, treatment and prevention of infections, it is necessary to differentiate between acute infections with primarily planktonic microorganisms and biofilm infections with overweight of clusters of microbial cells [1–4]. Most microorganisms in a biofilm grow slowly with down-regulated virulence and are heterogeneously distributed. They are less susceptible to antibiotics compared with their planktonic counterpart and can often not be cleared by the immune system [5–7]. Biofilm related infections can be device-related biofilm infections, such as prosthetic joint infections, or native tissue infections e.g. chronic osteomyelitis and cystic fibrosis. The current most effective treatment of biofilm related infections is to remove the infected medical device and to debride the infected tissue in combination with antibiotic therapy [8]. However, treatment failure is often, and many novel antibiofilm candidates are under research such as quorum sensing inhibitors, biofilm matrix degrading enzymes, and antimicrobial peptides.

An early and correct diagnosis is necessary for proper antibiotic administration. The minimum inhibitory concentration (MIC) is defined as the lowest concentration of the antibiotics preventing visible bacterial growth, while minimum bactericidal concentration (MBC) is the lowest concentration required to kill the bacteria. MICs are used by diagnostic laboratories mainly to confirm resistance. Determination of MIC and MBC is based on planktonic cells, whereas the minimum biofilm eradication concentration (MBEC) is defined as the lowest concentration of antibiotic required to eradicate the biofilm. MBEC has not been implemented in the clinical setting yet, and the published MBEC data are often incomparable because of different experimental conditions. Tables 1–2 illustrate examples of two important biofilm pathogens *Staphylococcus aureus* and *Pseudomonas aeruginosa,* and their MIC and MBEC values determined in several studies. As shown in Tables 1– 2, tryptic soy broth (TSB) and cation-adjusted Mueller Hinton broth (CaMHB) media are often used in these studies. TSB is a complex nutrient-rich general-purpose medium, while CaMHB is recommended for MIC testing of non-fastidious organisms according to ISO standard 20776–1: 2006 and is the standard medium in clinical laboratories in the US and European committee on antimicrobial susceptibility testing. High throughput methods for MBEC determination are most frequently used including 96-well microtiter plate combined with crystal violet staining, the Calgary biofilm device (CBD), or its commercial version the MBEC™ Assay (Innovotech, Canada) [23]. As shown in Table 1-2, MIC values were similar for most of the studies. However, MBEC of vancomycin towards *S. aureus* varies from 1 to more than 8000 mg L^− 1^. Similarly, the MBEC of tobramycin towards *P. aeruginosa* varies from 2 to 2560 mg L^− 1^. This large discrepancy in MBEC values is surprising, especially in light of some studies using the same strain. We hypothesize that the different test parameters and lack of standardization contributed to the large disparity.

**Table 1:** MBEC values of vancomycin for *S. aureus* found in a few studies. Please note ATCC 29213 were tested in several studies with different MBEC values

*S. aureus* strains	MIC (mg L^− 1^)	Challenge medium	Biofilm age (h)	Treatment duration (hours)	Biofilm model	MBEC (mg L^− 1^)
ATCC 49230	2	TSB	24	24, 72, 120	96-well microtiter plate	> 8000, > 8000, 2000 [9]
ATCC BAA1556	2	TSB	24	24, 72, 120	96-well microtiter plate	> 8000, 8000, 2000 [9]
ATCC 6538P, MRSA 16	0.5, 1	Not mentioned	24	24	Beads	> 2000 [10]
ATCC 29213**,** UOC18	1–2	CaMHB	24	1–72	CBD	> 1024 [11]
ATCC 29213	1	CaMHB	24	overnight	CBD	> 1024 [12]
ATCC 29213	1	MHB	48	24	CBD	> 512 [13]
ATCC 35556	1	CaMHB	24	24	CBD	> 256 [14]
ATCC 29213, ATCC 33591, VRS5	1–2	CaMHB	24	24	CBD	> 128 [15]
B341002, B346846	0.5–1	CaMHB	24	overnight	CBD	128, 64 [16]
Clinical isolates	0.5–1	CaMHB	18	24	96-well microtiter plate	8–16 [17]
Clinical isolates	0.5–1	CaMHB	18	24	96-well microtiter plate	4–32 [18]
40 MRSA isolates	1	TSB	24	overnight	CBD	1–64 [19]

**Table 2:** MBEC values of tobramycin for *P. aeruginosa* in a few studies

Strains	MIC (mg L^−1^)	Challenge media	Biofilm inoculation (hours)	Treatment duration (hours)	Biofilm model	MBEC (mg L^−1^
ATCC 27853	0.25–16	CaMHB	24	1, 2, 4	96-well microtiter plate	160–2560 [20]
ATCC 27853	0.25	TSB	24	24, 72, 120	96-well microtiter plate	2000, ≤250, ≤250 [9]
Strain K (PAK)	Not tested	LB	72	18	96-well microtiter plate	200–1600 [21]
PAO1	< 2	CaMHB	6	16–20	CBD	64 [22]
ATCC 27853	0.5	CaMHB	24	overnight	CBD	2 [12]

Biofilm infections such as prosthetic joint infections and chronic osteomyelitis are difficult to treat by oral or parenteral antibiotic therapy alone and debridement is needed for physical removal of biofilms [24, 25]. Management of orthopedic infections often involves use of local antibiotic impregnated cement after debridement to eradicate the potentially remaining planktonic bacteria and residues of biofilms. However, the applied antibiotic dose is often based on personal experiences of the surgeon as no recommendations are available regarding the amount of antibiotics to be used for spacer impregnation [26]. Despite of application of high doses, re-infections occur at 19% of cases [27]. Ideally, MBEC should be determined to guide the amount of antibiotics to be impregnated in the cement.

The purpose of this study was to demonstrate the influences of biofilm age, growth media, and antibiotics exposure time on *S. aureus* and *P. aeruginosa* biofilm removal using vancomycin (up to 3000 mg L^− 1^) and tobramycin (up to 80 mg L^− 1^), respectively. These two antibiotics were chosen because they are recommended for serious and life-threatening infections caused by Gram-positive bacteria and Gram-negative bacteria. TSB and CaMHB were chosen enabling comparison with studies in Tables 1 and 2. Four biofilm-forming strains were selected for this study. *S. aureus* strains DSM 110939 [28] was isolated from prosthetic knee infection while *S. aureus* ATCC 49230 was originally from chronic osteomyelitis. Both infections are known to be associated with biofilms. *P. aeruginosa* strain PA14 is a well-known biofilm former [29] and *P. aeruginosa* ATCC 15442 is also known to form biofilms [30, 31]. In addition, we investigated the possibility of using simple in vitro biofilm test such as Calgary biofilm method as initial screening of antibiofilm product by testing OSTEOmycin™, an allograft bone product loaded with either vancomycin or tobramycin [32, 33]. The available release profile of the OSTEOmycin™ products [34] made it possible to estimate concentration.

## Results

All four tested strains in this study were found susceptible to the tested antibiotics. The vancomycin MIC for both *S. aureus* strains was determined to be 1.25 mg L^− 1^, which is lower than breakpoint (2 mg L^− 1^) for *S. aureus*. Likewise, the tobramycin MIC for both *P. aeruginosa* strains was 0.63 mg L^− 1^, which is lower than breakpoint (4 mg L^− 1^) for *P. aeruginosa* according to Clinical breakpoints – bacteria (v 9.0) in European Committee on Antimicrobial Susceptibility Testing (http://www.eucast.org/fileadmin/src/media/PDFs/EUCAST_files/Breakpoint_tables/v_9.0_Breakpoint_Tables.pdf).

### Influence of biofilm age

**Fig. 1:**
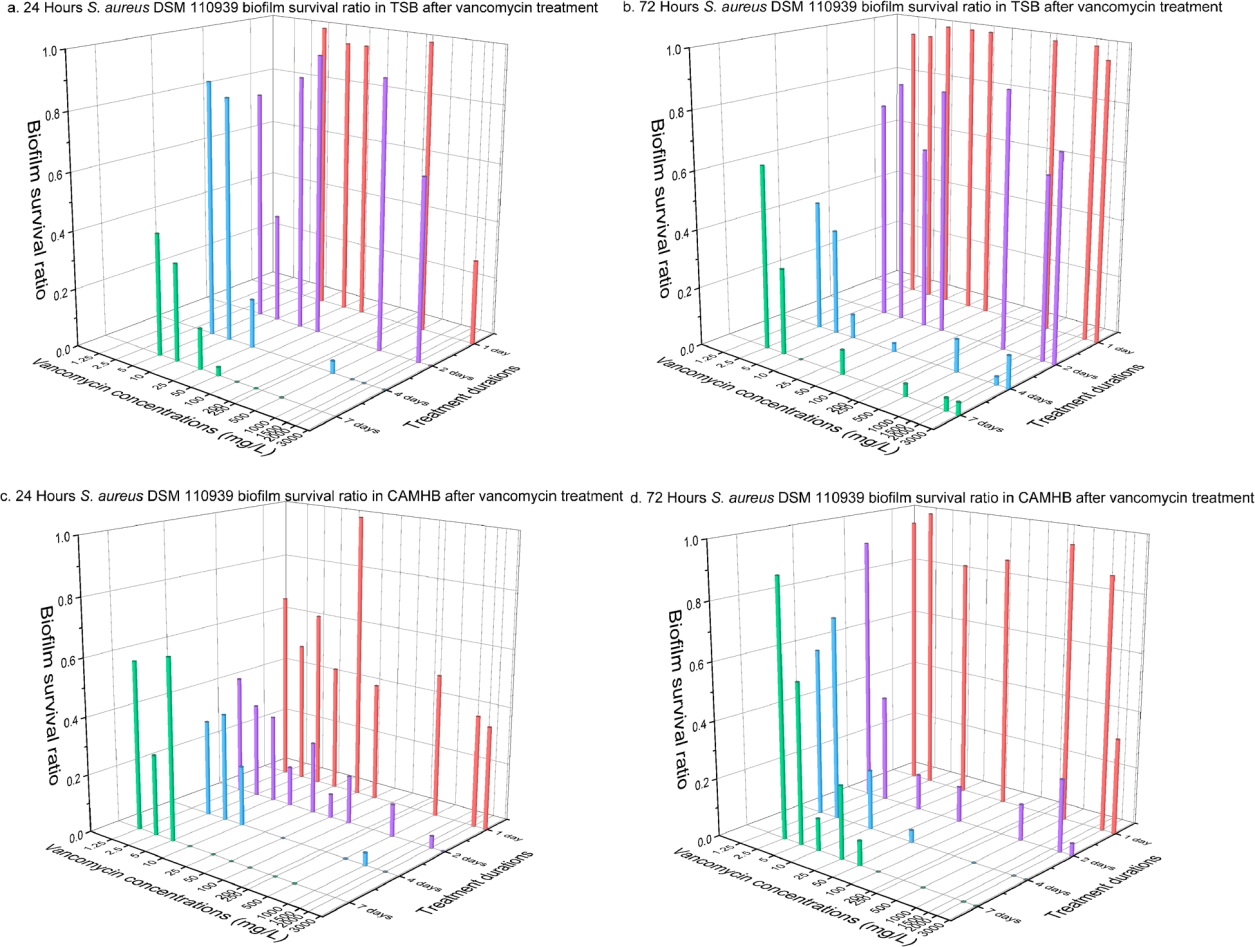
*S. aureus* DSM 110939 biofilm survival ratio after vancomycin treatment. Biofilms of *S. aureus* DSM 110939 were grown for 24 h or 72 h in TSB or CaMHB medium followed by vancomycin treatment for 1, 2, 4 or 7 days. Each data point contained at least 20 replicates conducted at two occasions

**Fig. 2:**
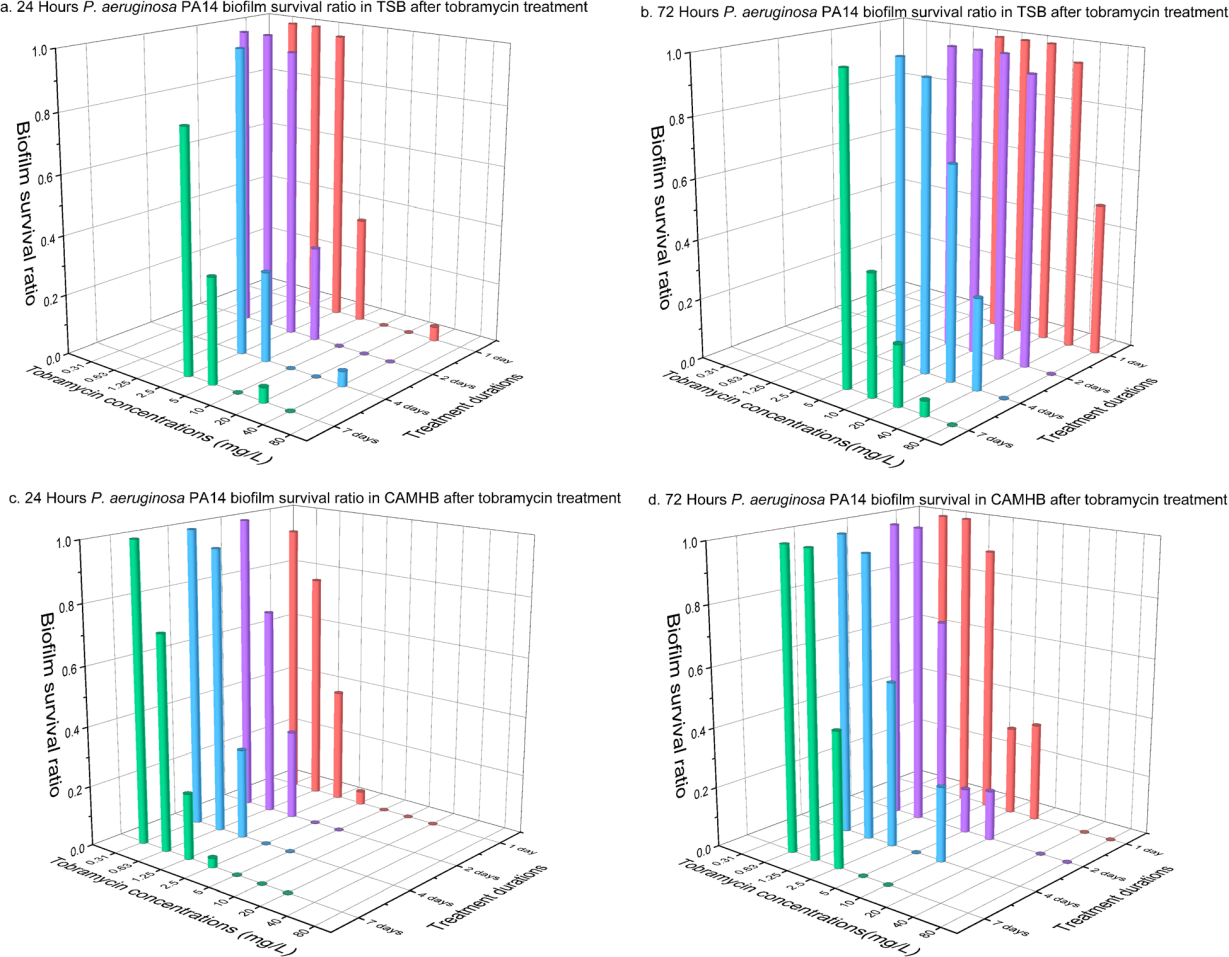
*P. aeruginosa* PA14 biofilm survival ratio after tobramycin treatment. Biofilms of *P. aeruginosa* PA14 were grown for 24 h or 72 h in TSB or CaMHB medium followed by tobramycin treatment for 1, 2, 4 or 7 days. Each data point contained at least 20 replicates conducted at two occasions

**Fig. 3:**
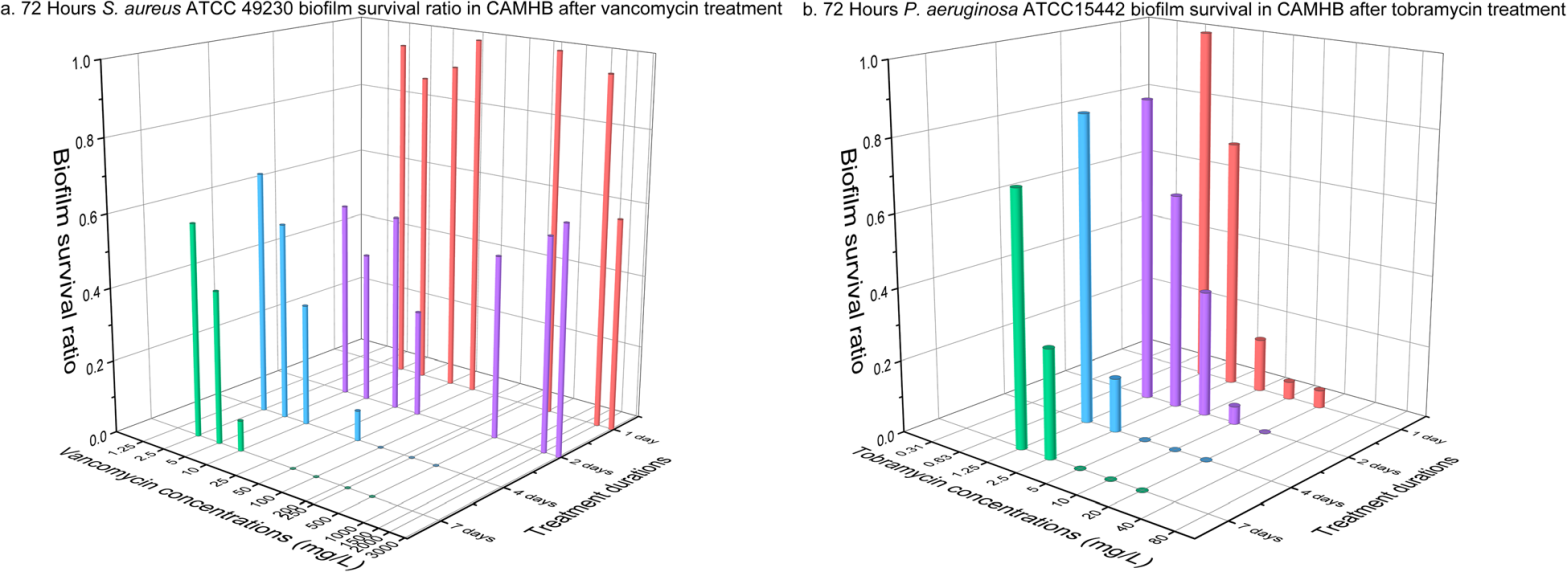
Survival ratio of 72 h *S. aureus* ATCC 49230 biofilms after vancomycin treatment (**a**) and 72 h *P****.*** *aeruginosa* ATCC 15442 biofilm after tobramycin treatment (**b**). Each data point contained at least 20 replicates conducted at two occasions

Biofilm growth is dynamic and mature biofilms are thought to be more antibiotic tolerant. In this study biofilms grew for 24 h or 72 h first and then were subjected to antibiotics challenge for different duration. It was found that the number of colony forming units (CFUs) were higher for 72 h biofilms than for 24 h biofilms by up to 1-log difference (*P* < 0.01, Figures S1 and S2). Additionally, 72 h biofilms were more difficult to eradicate than 24 h (*P* < 0.001) as shown in Figs. 1 and 2. It is important to stress that each data point in Figs. 1, 2 and 3 represents results for a minimum of 20 replicates from two independent experiments. Instead of MBEC value which defines complete killing of biofilms, biofilm survival ratio was chosen to present the percentage of replicates survived after treatment. The reason is that biofilm eradication was different among the replicates and a single MBEC value could not provide the information.

For complete killing of 24 h DSM 110939 biofilms in TSB medium, i.e. MBEC, exposure of the biofilms with a minimum of 1000 mg L^− 1^ of vancomycin for 4 days or 100 mg L^− 1^ for 7 days was required (Fig. 1a), whereas some 72 h biofilms still survived even with 3000 mg L^− 1^ of vancomycin after 7 days (Fig. 1b). In CaMHB medium it required 10 mg L^− 1^ of vancomycin for 7 days to remove 24 h DSM 110939 biofilms (Fig. 1c) and 10-fold more for 72 h biofilms (Fig. 1d).

In the case of strain PA14, a minimum of 10 mg L^− 1^ of tobramycin killed almost all 24 h biofilms in TSB media regardless of exposure duration (Fig. 2a) while for 72 h biofilms 80 mg L^− 1^ of tobramycin for at least 2 days was needed (Fig. 2b). In CaMHB medium, complete killing of 24 h biofilms was achieved with 5 mg L^− 1^ of tobramycin regardless of exposure duration (Fig. 2c), while it required more than 10 mg L^− 1^ for 72 h biofilms when the treatment was shorter than 7 days (Fig. 2d).

### Media

Biofilm formation depends on many factors including nutrient availability. The main nutrients in both TSB and CaMHB media are amino acids. In addition, TSB contains glucose (2.5 g L^− 1^) while CaMHB has starch (1.5 g L^− 1^). The number of CFUs in the biofilms growing in these two media were different (*P* < 0.05, Figure S1 and S2). On average, slightly more CFUs were found in biofilms growing in CaMHB than TSB, except 72 h PA14 biofilms.

When challenged with antibiotics, biofilms were more difficult to kill in TSB than in CaMHB. For 24 h DSM 110939 biofilms (Fig. 1a and c), seven-day treatment with 100 mg L^− 1^ and 10 mg L^− 1^ of vancomycin were required to kill all biofilms in TSB and CaMHB media, respectively. For 72 h DSM 110939 biofilms, none of the vancomycin treated achieved complete killing in TSB medium, while 100 mg L^− 1^ of vancomycin removed all biofilms after 7 days exposure in CaMHB (Fig. 1b and d). For all four treatment duration, 24 h PA14 biofilms, four-fold more tobramycin was needed in TSB than in CaMHB (10 and 2.5 mg L^− 1^, respectively) for near complete killing (Fig. 2a and c), while for 72 h biofilms, two-fold more tobramycin was required (80 mg L^− 1^ in TSB and 40 mg L^− 1^ in CaMHB) (Fig. 2b and d).

### Antibiotics exposure time

Extending vancomycin exposure time from 1 to 4 days reduced survival ratio of DSM 110939 biofilm in TSB (Fig. 1a and b, Table 3) and CaMHB media (Fig. 1c and d, Table 3). Prolonging treatment from 4 to 7 days showed no further killing except 24 h biofilms in TSB (Table 3). Increased killing by prolonging vancomycin exposure was also found for *S. aureus* ATCC 49230 biofilms (Fig. 3a).

**Table 3:** *P*-values for difference between *S. aureus* DSM 110939 biofilm survival ratio after vancomycin treatment of different durations. ** indicates *P* < 0.001

		TSB				CaMHB			
Biofilm age (hours)	Treatment durations (days)	1	2	4	7	1	2	4	7
24	1	–	**	**	**	–	**	**	**
	2		–	**	**		–	0.110	0.251
	4			–	**			–	0.790
	7				–				–
72	1	–	**	**	**	–	**	**	**
	2		–	**	**		–	0.220	0.027
	4			–	0.578			–	0.391
	7				–				–

In contrast to vancomycin, tobramycin is known to exhibit concentration-dependent bactericidal activity [35]. Removal efficacy of 24 h *P. aeruginosa* PA14 biofilm was not enhanced when duration was extended (Fig. 2a and c, Table 4). However, increasing killing was indicated for 72 h PA14 biofilms (Fig. 2b and d, Table 4) as well as for 72 h *P. aeruginosa* ATCC 15442 biofilms (Fig. 3b).

**Table 4:** *P*-values for difference between *P. aeruginosa PA14* biofilm survival ratio after tobramycin treatment of different durations. * indicates *P* < 0.01 and ** indicates *P* < 0.001

		TSB				CaMHB			
Biofilm age (hours)	Treatment durations (days)	1	2	4	7	1	2	4	7
24	1	–	0.553	0.188	0.578	–	0.042	0.535	0.309
	2		–	0.220	0.518		–	*	0.218
	4			–	0.101			–	0.113
	7				–				–
72	1	–	*	**	**	–	0.016	**	**
	2		–	**	**		–	0.128	*
	4			–	**			–	0.233
	7				–				–

### Strains

The two *S. aureus* strains have the same vancomycin MIC value. Although the necessary concentration of vancomycin for biofilm eradication differed slightly, the same tendency is indicated for both strains that prolonged vancomycin treatment eradicated more biofilms. Similarly, the two *P. aeruginosa* strains have the same tobramycin MIC value and extended tobramycin treatment lowered MBEC values for 72 h biofilms for both strains.

### OSTEOmycin™

Both 72 h and 168 h biofilms were challenged with OSTEOmycin™ for one, two, four, and seven days (in total eight conditions). All PA14 biofilms were cleared after 1 day exposure to OSTEOmycin T™ and remained sterile after 7 days exposure. All *S. aureus* ATCC 49230 biofilms were eradicated except that three replicates of 72 h biofilms survived 2 days treatment.

## Discussion

### Biofilm age

Several biofilm models have been developed, each with many experimental parameters that can be adjusted. This flexibility inevitably makes it difficult to compare results obtained with varying conditions chosen in different studies. In the current study we confirmed previous findings that mature biofilm have reduced antibiotics susceptibility compared with young biofilms [36–39]. However, no definition of young and mature biofilm has been universally adopted. In the case of *P. aeruginosa*, some considered 4 h biofilm as young and 24 h as mature [40], while others considered 24 h as young and 12 days biofilm as mature [38]. Similarly, 6 h *S. aureus* biofilm was considered as young and 24 h as mature [41], whereas some considered 7 days old biofilm as mature [42]. The inconsistency in the different biofilm studies underlines the need for a form of consensus definition and a simple way to measure maturity. The textbook version of biofilm formation involves bacterial initial attachment to a solid surface, the formation of microcolonies on the surface, and finally differentiation of microcolonies into exopolysaccharide-encased, mature biofilms. However, studies often assume the maturity of the biofilm without looking into the structure of the biofilms or even CFUs of biofilm. In the case of MIC testing, a crucial parameter is inoculum size which is set to be 5 × 10^5^ CFU mL^− 1^. It is because MIC values can increase concurrently with increasing number of CFUs [43].

The current study treated 24 h biofilms as young and 72 h as mature. The CFU per biofilm shown in Figure S1 and S2 indicated continuous growth in cell number after 24 h for up to 1-log. In batch culture, bacterial growth curve defines the different stages of planktonic culture growth. Similarly, the biofilm formation curves can be established for each strain and growth condition. It was shown previously [12] that using CBD the number of *E. coli* ATCC 25922 and *P. aeruginosa* ATCC 27853 continuously increased over 24 h while the growth of *S. aureus* stagnated after 7 h under the same condition. As the growth phase of the biofilm influences antimicrobial susceptibility, it is therefore important to construct the biofilm growth curve for each strain under the chosen conditions.

### Growth media

Biofilm eradication was found different with the two media (Fig. 1 and Fig. 2). Different composition of media is reported to change the activity of antibiotics [44–46]. The Ca^2+^ and Mg^2+^ ions in CaMHB media are required for a correct antimicrobial susceptibility testing because those ions reflect the divalent cation concentration in human blood [43, 47–49]. Neither of the two tested media, TSB and CaMHB, resembles in vivo conditions. However, use of CaMHB makes it possible to compare with MIC results, while TSB has been frequently used in other publications (Tables 1 and 2). Other media such as brain-heart infusion broth [50], TSB supplemented with glucose [51], LB [21], and chemically defined media such as basal medium 2 and M9 minimal media [52] have also been used in studies. The choice of media is known to affect biofilm formation [53, 54], but a standardized medium to assess the activity of antibiofilm agents has not been established. It is difficult to standardize because the in vivo environment of biofilm infections varies depending on the location of the infection, hence the optimal medium should be developed for each infection, for example, medium supplemented with mucin for studying cystic fibrosis lung infection [55], or saliva containing medium for studying oral biofilms [56], or human urine for urinary tract infections [57, 58]. Besides nutrient source, in vivo conditions are far more complex with presence of immune systems and varying oxygen level etc., the antibiotics concentration needed for biofilm eradication will most likely be different from in vitro results. For comparison across different studies, a simple and widely available culture medium is suitable, but for estimation of in vivo biofilm killing host factors in form of, for example, serum, plasma, or blood should be included in testing medium.

### Antibiotics exposure time

Vancomycin displayed a time-dependent eradication of *S. aureus* biofilms (Table 2) which has been demonstrated in other studies [42, 59, 60]. Post et al. have shown continuous reduction of viable *S. aureus* biofilm cells over 28 days [42]. This indicates that further killing could be possible by prolonging the antibiotic exposure time in the current study and complete eradication could be achieved at lower vancomycin concentration.

In contrast to vancomycin, tobramycin exhibits concentration-dependent activity [61–64]. The current study indicated that tobramycin displayed concentration-dependent activity for 24 h PA14 biofilms. However, increased killing of 72 h biofilms were observed with prolonged exposure. Castaneda et al. found increased biofilm antimicrobial susceptibility with increasing antimicrobial exposure time including tobramycin against *P. aeruginosa* biofilms [9], whereas Walters et al. only found little reduction in *P. aeruginosa* biofilm cell count with longer tobramycin treatment [65]. Futures studies are needed to investigate the time-dependency of tobramycin antibiofilm effect.

Regardless of the antimicrobials being time-dependent or concentration-dependent on planktonic bacteria, it may be different on biofilm cells because of the presence of biofilm matrix. Exposure time may play an important role in determination of killing effect, because the biofilm matrix may slow down antimicrobial penetration [66]. Therefore, a killing curve is much more informative than a definitive MBEC value determined at a fixed time point.

### OSTEOmycin™

Since the antibiotic concentration needed for biofilm eradication is far above the parenterally administrated levels, local delivery of antibiotics may achieve concentrations high enough for biofilm killing. In this study, OSTEOmycin™ showed a strong biofilm eradication efficacy and completely removed biofilm in all tested conditions except three 72 h *S. aureus* biofilms. OSTEOmycin™ is a product developed based on Winkler et al. 2000 [34]. According to the study, 1 g human cancellous bone impregnated with vancomycin released around 20,000 mg L^− 1^ vancomycin in 3 mL of 5% human albumin solution after 1 day and decreased to around 100 mg L^− 1^ after 7 days. Accordingly, it implies that approximately 16,800 mg L^− 1^ of vancomycin after 1 day and 84 mg L^− 1^ after 7 days were released with the applied amount in this study. When impregnated with tobramycin, it released more than 10,000 mg L^− 1^ tobramycin after 1 day and decreased to around 30 mg L^− 1^ after 7 days [34], suggesting 6600 mg L^− 1^ of vancomycin after 1 day and 19.8 mg L^− 1^ were released after 7 days with the applied amount in this study. These concentrations are much higher than the MBEC values found in Figs. 1 and 2, which likely explains the high efficacy. This indicates that prolonged antibiotics treatment may not be necessary when sufficiently high concentration of antibiotics is administered in the beginning of treatment. OSTEOmycin was also shown to be efficient for local treatment of osteomyelitis in the clinic although recurrence may still occur in complex cases within an unknown period of time [33]. The limitation of this study is that OSTEOmycin™ was not tested in a medium resembling the nutrient composition in the bone under in vivo like conditions, and more clinically used or candidate antibiofilm products for osteomyelitis could have been evaluated, such as antibiotics impregnated cement or hydrochlorous acid. Ideally, the in vitro effect of these antibiofilm products could be compared to clinical outcome to validate the assay. Assays developed in such as way could be used to guide the dose of antibiotics for clinical application.

In this study, the used conditions (nutrient rich media, pH, atmospheric oxygen level, shear, biofilm growth in static system, mono species biofilm etc.) were not specific for a distinct biofilm infection and more suitable for initial testing of antibiofilm product. It was by no means meant as a standardization or guideline for clinical application. The purpose was to raise the awareness that biofilm eradication depends on many factors, including the ones mentioned here, but also pH, oxygen level, temperature, shear, and complicated by polymicrobial community interactions and the presence of human factors such as the human immune systems. For specific biofilm infections, we think it is necessary to develop assays with in vivo like environment and validate obtained results by comparing with clinical outcome.

## Conclusion

This study showed biofilm removal efficacy was influenced by media, biofilm age and antibiotics treatment duration. It is therefore necessary to take these parameters into consideration when designing experiments. We recommend choosing the conditions most similar to the in vivo situation and explaining the rationale when reporting. This study also showed that in vitro biofilms were possible to be eradicated when treated with long-term high concentrations of antibiotics. This finding needs to be confirmed by in vivo studies.

## Methods

### Bacterial strains, growth media and antibiotics

*S. aureus* strains DSM 110939 [28] and ATCC 49230 were tested with vancomycin (Sigma-Aldrich). *P. aeruginosa* strains PA14 and ATCC 15442 were tested with tobramycin (Sigma-Aldrich). Both tryptic soy broth (TSB) (Sigma-Aldrich) and cation-adjusted Mueller Hinton broth (CaMHB) (Sigma-Aldrich) media were employed in susceptibility testing.

### Minimum inhibitory concentration (MIC) determined by the broth microdilution method

The broth microdilution method was used to determine the MIC of each strain according to the procedures described in Wiegand et al. [43]. Briefly, each strain was inoculated on TSB agar plate for 24 h. Then five well-isolated colonies were selected and inoculated in a 50 mL tube with 20 mL CaMHB until the OD600 value of the culture reached around 0.6. The culture was diluted to approximately 1 × 10^6^ colony-forming unit (CFU) mL^− 1^. Then, 100 μl of the diluted culture was added into each well of a 96-well-plate containing 100 μl of antibiotics at concentrations from 0.31 to 80 mg L^− 1^ of tobramycin or from 1.25 to 3000 mg L^− 1^ of vancomycin). The plate was covered and inoculated at 37 °C with shaking at 150 rpm for 24 h. After that, OD595 of each well was measured by Infinite F200 Pro (Tecan Group Ltd., Switzerland) to determine MIC.

### Biofilm antibiotics susceptibility testing by Calgary biofilm device (CBD)

CBD [67] was used to grow biofilms. An illustration of the experimental procedure is given in Figure S3. Briefly, biofilms were formed by immersing the pegs of a microtiter lid (Nunc™ 445497) into the biofilm growth microtiter plate, 150 μl of the diluted culture containing 10^4^ CFU was added into the wells of 96 well microtiter plate (Thermo Fisher Scientific) and then covered with peg lid The biofilms were allowed to grow in TSB or CaMHB media at 37 °C with shaking at 150 rpm for 24 h or 72 h. After incubation, the lid with biofilms was transferred to a rinse plate containing 200 μl saline in each well and incubated for 1 min. The rinsed lid was then transferred to a challenge plate containing 200 μl antibiotics solution in each well. The antibiotics were prepared in the media used for growing biofilms. The plates were challenged for 24, 48, 96 or 168 h at 37 °C with shaking at 150 rpm. After challenged in the antibiotics solution, the lid containing biofilms was rinsed twice with fresh saline each time and then transferred to a recovery plate containing 200 μl sterile media followed by sonication at 40KHz for 5 min.

After removal of the lid, the recovery plate was inoculated for another 24 h at 37 °C with shaking at 150 rpm and OD595 measured by Infinite F200 Pro to determine the biofilm removal efficacy. All tests were repeated at least on two occasions with minimum 10 replicates each time. Percentage of the surviving replicates was calculated and presented as biofilm survival ratio.

### Biofilm eradication by OSTEOmycin™

OSTEOmycin™ samples were obtained from European Cell and Tissue Bank. Two clinical strains *S. aureus* ATCC 49230 and *P. aeruginosa* PA14 were chosen for this test. Seventy-two hours (3-days) or 168 h (7-days) biofilms were challenged with OSTEOmycin™ for 1, 2, 4 or 7 days following the method described above. *S. aureus* ATCC 49230 biofilms were challenged with 280 g L^− 1^ OSTEOmycin V™ in CaMHB, while *P. aeruginosa* PA14 biofilms were subjected to 220 g L^− 1^ OSTEOmycin T™.

### Statistics analysis

ANOVA was used to calculate the difference between biofilm formation on CBD pegs. Binary logistic regression model was used to compare biofilm removal efficacy under different conditions.

## Supplementary information

**Additional file 1: Figure S1**. *S. aureus* biofilm formation on CBD. After 24 or 72 h of growth, biofilms were removed from the pegs, transferred into the recovery plate and harvested by sonication. Six random wells of each row were selected for CFU count, in total 48 wells per plate. The number of CFUs per peg were different under different conditions. Generally 3 days incubation resulted in more CFUs per peg. **Figure S2**. *P. aeruginosa* PA14 biofilm formation on CBD. After 24 or 72 h of growth, biofilms were removed from the pegs, transferred into the recovery plate and harvested by sonication. Six random wells of each row were selected for CFU count, in total 48 wells per plate. The number of CFUs per peg were different under different conditions. Generally 3 days incubation resulted in more CFUs per peg. **Figure S3.** Flow diagram of the MBEC assay.

## Data Availability

The datasets used and/or analysed during the current study are available from the corresponding author on reasonable request.

## Abbreviations

CaMHB: Cation-adjusted Mueller Hinton broth
CBD: Calgary biofilm device
CFU: Colony forming units
MBEC: Minimum biofilm eradication concentration
MBC: Minimum bactericidal concentration
MIC: Minimum inhibitory concentration
TSB: Tryptic soy broth
