# Supplementary material for: Influence of biofilm growth age, media, antibiotic concentration and exposure time on Staphylococcus aureus and Pseudomonas aeruginosa biofilm removal in vitro
Source: BMC Microbiol. 2020 Aug 24;20:264. doi: 10.1186/s12866-020-01947-9 (PMC7444035; doi:10.1186/s12866-020-01947-9)
Supplement: Supplementary file 1 — Additional file 1: Figure S1. S. aureus biofilm formation on CBD. After 24 or 72 h of growth, biofilms were removed from the pegs, transferred into the recovery plate and harvested by sonication. Six random wells of each row were selected for CFU count, in total 48 wells per plate. The number of CFUs per peg were different under different conditions. Generally 3 days incubation resulted in more CFUs per peg. Figure S2. P. aeruginosa PA14 biofilm formation on CBD. After 24 or 72 h of growth, biofilms were removed from the pegs, transferred into the recovery plate and harvested by sonication. Six random wells of each row were selected for CFU count, in total 48 wells per plate. The number of CFUs per peg were different under different conditions. Generally 3 days incubation resulted in more CFUs per peg. Figure S3. Flow diagram of the MBEC assay. [file 12866_2020_1947_MOESM1_ESM.docx]

Supplemental Materials

**Figure S1 *S. aureus* biofilm formation on CBD.** After 24 or 72 hours of growth, biofilms were removed from the pegs, transferred into the recovery plate and harvested by sonication. Six random wells of each row were selected for CFU count, in total 48 wells per plate. The number of CFUs per peg were different under different conditions. Generally three days incubation resulted in more CFUs per peg.

**
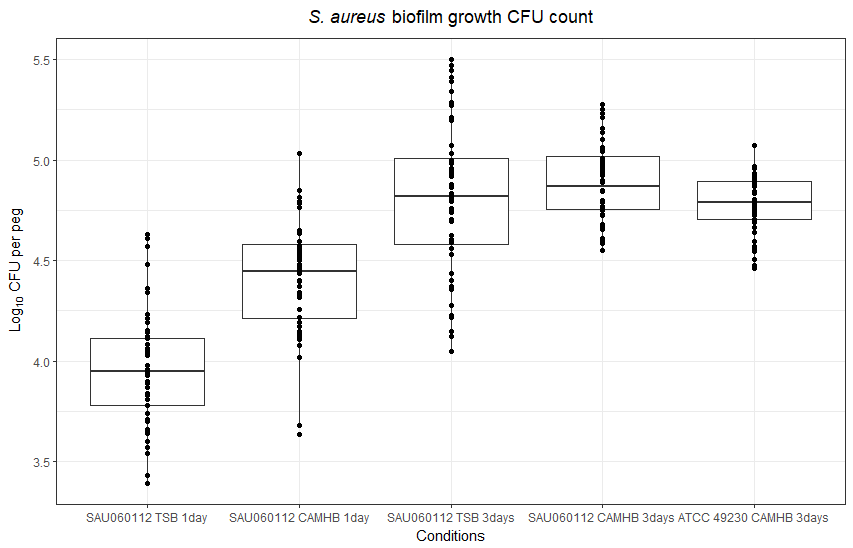
**

**Figure S2 *P. aeruginosa* PA14** **biofilm formation on CBD.** After 24 or 72 hours of growth, biofilms were removed from the pegs, transferred into the recovery plate and harvested by sonication. Six random wells of each row were selected for CFU count, in total 48 wells per plate. The number of CFUs per peg were different under different conditions. Generally three days incubation resulted in more CFUs per peg.


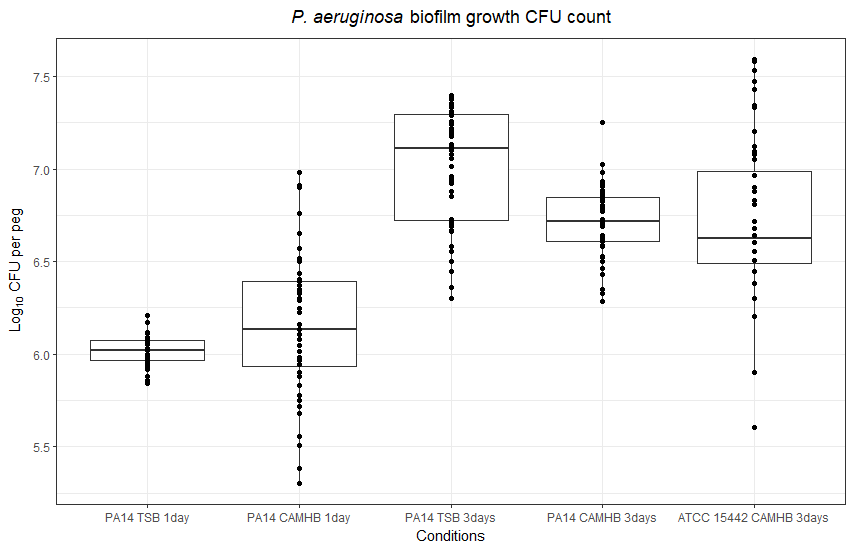


**Figure S3 Flow diagram of the MBEC assay.**

**
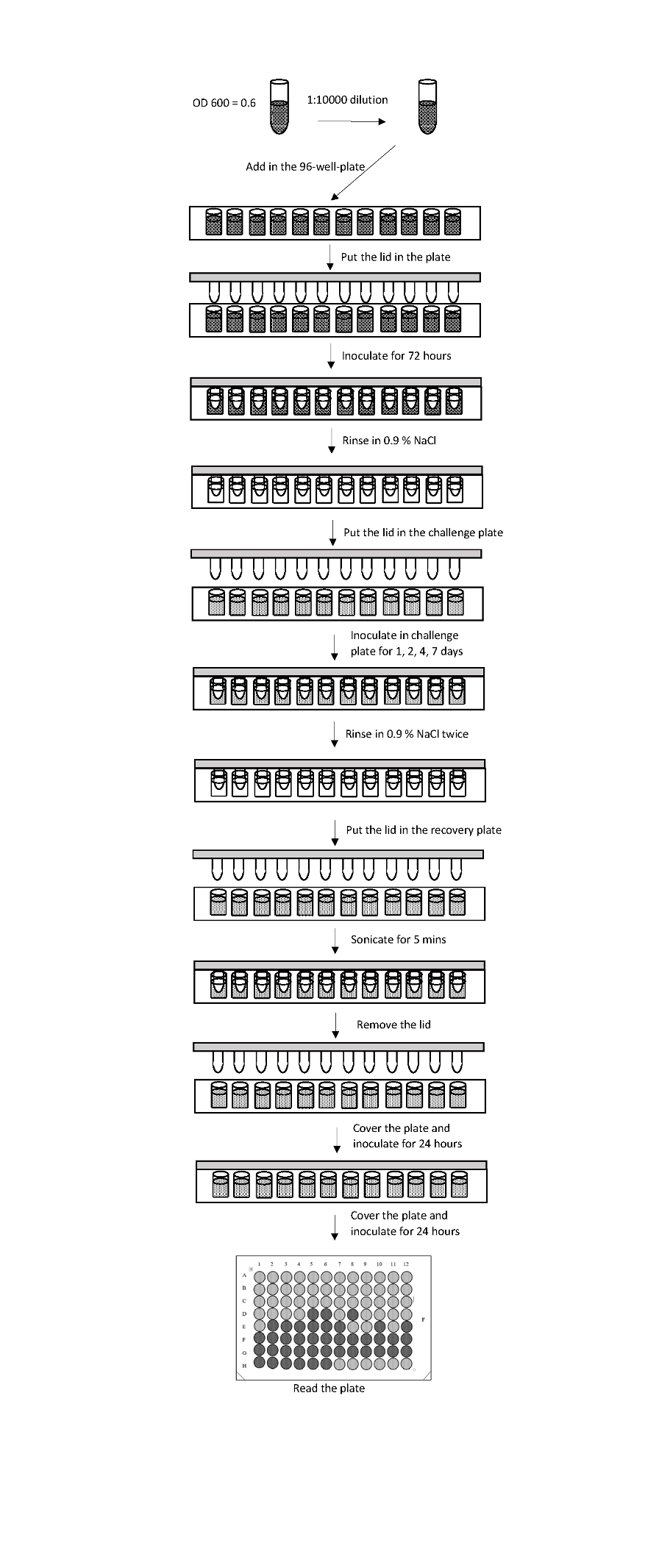
**
